# New forms of checks and balances are needed to improve research integrity

**DOI:** 10.12688/f1000research.3714.1

**Published:** 2014-05-28

**Authors:** Elizabeth Iorns, Christin Chong

**Affiliations:** 1 Science Exchange Inc., 555 Bryant Street, #939, Palo Alto, CA, 94301-1704, USA; 2 Department of Neurology, University of California San Francisco, 1550 4th St #546A, San Francisco, CA, 94158-2324, USA

## Abstract

Recent attempts at replicating highly-cited peer-reviewed studies demonstrate that the “reproducibility crisis” is indeed upon us. However, punitive measures against individuals committing research misconduct are neither sufficient nor useful because this is a systemic issue stemming from a lack of positive incentive. As an alternative approach, here we propose a system of checks and balances for the publishing process that involves 1) technical review of methodology by publishers, and 2) incentivizing direct replication of key experimental results. Together, these actions will help restore the self-correcting nature of scientific discovery.

## Introduction

The scientific method provides a systematic framework for formulating, testing and refining hypotheses. By definition, it requires findings to be reliable so that theories can be refined and scientific progress can occur. Recently, it has become clear that the scientific method as it is currently being practiced is failing in self-correction, with multiple studies indicating that more than 70% of surveyed peer-reviewed articles cannot be independently verified ^1– 4^. Unfortunately, instead of focusing on new systems to promote high quality reproducible research, most resources and attention are focused on trying to police the scientific community by investigating allegations of research misconduct. This approach is destined to fail, because the problem is systemic and not caused by a few bad players who can be caught and punished. From 1994–2003, 259 cases of misconduct were formally investigated by the Office of Research Integrity ^5^. In contrast, ~480,000 papers funded by the NIH were published ^6^. It would be impractical and ineffective to investigate why 70% of published findings are irreproducible, even though ultimately the ability to repeat and build upon prior work is the key component of research integrity that we should care about. Instead, truly addressing the “reproducibility crisis” requires establishing new checks and balances for the publishing process through 1) technical review of methodology by publishers, and 2) incentivizing direct replication of key experimental results. If we, the scientific community, fail to ensure the quality of the research we produce, other parties with their own vested interests will step in to police us instead ^7^.

### 1. Checks: Publishers need to verify quality of research through third-party technical review

Publishers are uniquely placed to significantly improve reproducibility because of their inherent need to garner respect from the scientific community. *Nature* and EMBO are two stand-out examples who are leading the way on ensuring the quality of the research published in their journals. Moreover, current efforts to ensure quality using peer-review alone to weed out irreproducible research are not effective. One reason is that the breadth of technical knowledge that is now required to review a single study is beyond individual scientists. The number of authors per article has increased over the last decade ^8^. In contrast, peer review still relies on two or three peers who are unlikely to be qualified to assess every experimental technique in the study. *Nature* has implemented an impressive new policy to reduce irreproducibility of its published papers ^9^, and a key aspect to this is employing expert statisticians to review the statistical analysis of papers. Currently, a major limiting factor for implementing technical review is the lack of standardization for methodology design and required controls. Establishing and implementing these standards to ensure the technical quality of the research published in their journals is an effective value-added service that publishers should provide as a separate power in the scientific community. The Resource Identification Initiative ( https://www.force11.org/node/4463 date accessed: 2014-04-24) is an example of practical implementation for reporting of materials and methods in a standardized and machine-readable manner. Similar to successful mandates on open access to raw data, journals wield the power to require clear methodology as prerequisite for publication. Further, analogous to open data, the nascent implementation of standardized methodologies will likely yield debates, but lively discussions by the scientific community are useful for policy refinement ( http://blogs.plos.org/everyone/2014/03/08/plos-new-data-policy-public-access-data/ date accessed: 2014-04-25).

### 2. Balances: Direct replication needs to be incentivized for science to be self-correcting

While journals should carry technical review responsibilities, establishing positive incentive structures for reproducible science is necessary to balance the pressure of producing high-profile publications at all costs. Of course, there will always be edge cases where it is not practical to directly replicate findings (for example unpredictable or one-off events like an earthquake), but for the majority of findings it should be possible to *directly* replicate them. That is, repeat the experiment as-is, while collecting additional information such as “ *the reliability of the original results across samples, settings, measures, occasions, or instrumentation*” ^10^. This is separate from *conceptual* replication, which is “an attempt to validate the *interpretation* of the original observation by manipulating or measuring the same conceptual variables using different techniques” ^10^. It is also separate from re-analysis of existing raw data to check for errors in analysis and presentation, but where no new data are obtained. Therefore, directly reproducing experiments is not merely redundant effort, because new data are generated and analyzed to demonstrate the robustness of the original results.

Journals such as *F1000Research* and PLOS ONE ( http://f1000research.com/author-guidelines, http://www.plosone.org/static/publication, date accessed: 2014-03-14) now consider direct replication of original studies, but even a place to publish is not sufficient because there needs to be an effective system to incentivize scientists to conduct replication studies in the first place. The simplest way to conduct replication studies is via fee-for-service technical providers because of their pre-existing methodological expertise and neutral academic involvement (i.e. they are motivated by an operational or a monetary incentive, and thus do not fear retribution from their peers or have the need to accumulate high impact ‘novel’ publications). Similarly, grants specifically designated for research integrity are vital for driving replication ( http://www.arnoldfoundation.org/reproducibility-initiative-receives-13m-grant-validate-50-landmark-cancer-studies date accessed: 2014-04-28). These are strategies used by the Reproducibility Initiative ( https://www.scienceexchange.com/reproducibility, date accessed: 2014-03-14), and it remains to be proven whether it will be a cost-effective mechanism to conduct direct replications.

The recent ascent of crowd-sourced post publication peer reviews have identified manuscripts with problematic content, but they remain most active for articles on new techniques that other researchers are eager to replicate for their own experiments (e.g. http://www.ipscell.com/stap-new-data/ date accessed: 2014-04-28 and http://f1000research.com/articles/3-102/v1 date accessed: 2014-05-20). Therefore, positively incentivizing direct replication is necessary for science to become self-correcting again, because no one would selectively publish only their experiments that worked or manipulate their findings knowing that a replication attempt, whether experimental or analytical, would not find the same significant outcome. Scientists would also be more willing to share their raw data and full methodologies before publishing because they want to make sure that their findings are reproducible. Not identifying robust and reproducible research is very costly and impairs our ability to make effective progress against diseases like cancer in which we have already invested billions of dollars. Establishing new checks and balances with existing members of the scientific community such as publishers and fellow scientists is infinitely more preferable than those imposed by outside authorities. And if science progresses by “standing on the shoulders of giants”, it is our duty as scientists to ensure that the “shoulders” are steadfast for our peers.
