# Supplementary material for: New forms of checks and balances are needed to improve research integrity
Source: F1000Res. 2014 May 28;3:119. [Version 1] doi: 10.12688/f1000research.3714.1 (PMC4197740; doi:10.12688/f1000research.3714.1)
Supplement: Peer review history

# Peer review: Referee response for version 1

**DOI:** 10.5256/f1000research.3980.r4918

**Published:** 2014-06-18

**Authors:** David Soll

**Affiliations:** 1 Department of Biology, University of Iowa, Iowa City, IA, USA

Iorns and Chong state in the first paragraph of their Opinion Article that “ *70% of surveyed peer-reviewed articles cannot be independently verified*”. Iorns, who heads the company, Science Exchange, Inc., reported the same statistic in an interview with Jennifer Welsh in Business Insider, 2012. Now she and Christin Chong present a set of recommendations for alleviating this problem. But the way they support their claim that 70% of research is irreproducible is problematic. They base their value primarily on four references ^1^ ^,^ ^2^ ^,^ ^3^ ^,^ ^4^ that demand scrutiny. These references include three cases on drug effects that are marginal and a fourth on sex differences. Two of the references include data not peer reviewed and authored by individuals from commercial companies ^1^ ^,^ ^2^. A third is retrospective and involves the re-evaluation of statistical calculations of the original authors ^3^. Only one, testing the effects of drugs on increased longevity of SOD1G934 mice, provides data that can be assessed ^4^ , and even those data, obtained in an impressive manner, are presented in a review article.

There is merit in questioning the reproducibility of studies on marginal drug effects or sex differences, but it seems irresponsible to present, as Iorns and Chong have, a sweeping statement that 70% of all published peer-reviewed articles are irreproducible, even with the qualification of “surveyed” articles. Do these authors really believe that this 70% value applies to studies on signal transduction pathways, the phenotypes of mutants from viruses to bacteria to mammals, the interactions and roles of cytoskeletal molecules, the molecular evolution of species, the functions of molecules in embryogenesis and a vast variety of other biological fields? If Iorns and Chong had limited their commentary to the efficacy of drugs in model systems with marginal effects, they could have made an important and plausible case. But even then they would have had to do a better job referencing their argument. And to bring up the fact that 259 cases of misconduct were investigated by the Public Health Service, followed by their statement “ *That in contrast ~480,000 papers funded by the NIH were published*.”, appears to be an attempt to globalize the problem by insinuation rather than hard supporting data.

The suggestion by the authors that publishers should assess the methods and statistics used by third parties is already in place. It is, obviously, the peer review system, and of course it has its problems. But the insinuation is that this process is failing in 70% of cases. Publishers should indeed be more responsible for making sure that reviewers are selected who can really assess whether the methods employed and the statistics applied are valid, especially when marginal effects are claimed. I am sure that all other scientists would whole heartedly agree with that general suggestion. But a vehicle for immediately replicating data in every published paper is extraordinarily impractical, potentially very expensive and not at all necessary in areas of research in which answers are far-more straight forward. And who would foot the bill? The publishers? They are, in almost all cases, for-profit. For replication, they would charge a small fortune. And would scientists spend half of their research funds replicating other scientist’s discoveries. With the radical decrease in funding we are now experiencing, I would not bet on it. Iorns is co-founder of Science Exchange, Inc., a for profit company that  charges scientists to have measurements performed in 900 laboratories worldwide that appear to have been recruited to perform experiments for a fee, and a profit, presumably for them and  a presumable cut for Science Exchange, Inc. Would Science Exchange, Inc. be the vehicle for such testing?

The authors should realize that big discoveries are immediately reproduced by other scientists, to build on those discoveries. Therefore, most scientists are obsessed with the validity of their results. And reproducibility is a tough chore if scientists do not apply the exact same procedures, under the exact same conditions, with the exact same strains and the exact same reagents. Biological systems, from cell cultures to biofilms to biochemical reactions have inherent plasticity and variability, highly responsive to the smallest changes in genetic background, temperature, composition of the atmosphere, trace elements, source of reagents and extracts, and even the quality of double distilled water. But contradictions in the results published by different laboratories have a way of “shaking themselves out”. Most seasoned biologists at the bench know this is the case. Iorns and Chong have made a reasonable case for a limited area of biomedical research that involves searching for small or marginal effects and which involve apparently high noise levels. But they have presented no proof that supports their claim that 70% of all biomedical research is irreproducible, an overstatement which insinuates a significant number of scientists are at worst actively trying to dupe the rest of the scientific world or at best incompetent. By globalizing the problem to a majority of the entire scientific research community in the first paragraph of their commentary, they have sensationalized the targeted problem.

I have read this submission. I believe that I have an appropriate level of expertise to state that I do not consider it to be of an acceptable scientific standard, for reasons outlined above.

# Peer review: Referee response for version 1

**DOI:** 10.5256/f1000research.3980.r4917

**Published:** 2014-06-16

**Authors:** Ivan Oransky

**Affiliations:** 1 Department of Science, Health and Environmental Reporting, New York University, New York, NY, USA; 2 Retraction Watch, New York, NY, USA

Thank you for the opportunity to review this article. It makes an important argument in a critical area of inquiry, and deserves publication.

I have some specific suggestions for improvement below:

1. **" *...punitive measures against individuals committing research misconduct are neither sufficient nor useful because this is a systemic issue stemming from a lack of positive incentive.*"**
  I'd agree that such measures are not sufficient, but what is the evidence that they are not useful?
2. ***"From 1994–2003, 259 cases of misconduct were formally investigated by the Office of Research Integrity ^5^. In contrast, ~480,000 papers funded by the NIH were published ^6^. It would be impractical and ineffective to investigate why 70% of published findings are irreproducible, even though ultimately the ability to repeat and build upon prior work is the key component of research integrity that we should care about." ***
  While it is useful to discuss ORI's limited resources, there are more recent data on their investigations, for example Figure 4 of this paper: http://www.plosmedicine.org/article/info%3Adoi%2F10.1371%2Fjournal.pmed.1001563. I'd also make it clear that the ORI only has jurisdiction over fabrication, falsification, and plagiarism (FFP) – e.g. scientific misconduct -- and there is no evidence that FFP is responsible for most irreproducibility. So I wouldn't rely on ORI stats for why it's impractical and ineffective to investigate irreproducibility.
3. ***"...peer review still relies on two or three peers who are unlikely to be qualified to assess every experimental technique in the study."***
  I agree, but can the authors say more about how standardization of methodology design and required controls will solve this problem?
4. ***"The recent ascent of crowd-sourced post publication peer reviews have identified manuscripts with problematic content, but they remain most active for articles on new techniques that other researchers are eager to replicate for their own experiments (e.g. http://www.ipscell.com/stap-new-data/ date accessed: 2014-04-28 and http://f1000research.com/articles/3-102/v1 date accessed: 2014-05-20*).**"
  While these two examples demonstrate cases in which post-publication peer reviews are " *on new techniques that other researchers are eager to replicate for their own experiments,*" I'm not sure that's really where post-publication peer review is most active. I would mention PubPeer here, at the very least for context.
5. The authors make a few comments about costs, which are welcome: **". *..it remains to be proven whether it will be a cost-effective mechanism to conduct direct replications*."** and **" *Not identifying robust and reproducible research is very costly and impairs our ability to make effective progress against diseases like cancer in which we have already invested billions of dollars." ***It would be useful to try to estimate how much replication efforts will cost, and where this funding will come from.

I have read this submission. I believe that I have an appropriate level of expertise to confirm that it is of an acceptable scientific standard.

# Peer review: Referee response for version 1

**DOI:** 10.5256/f1000research.3980.r4922

**Published:** 2014-06-10

**Authors:** Andrew Chalmers

**Affiliations:** 1 Department of Biology and Biochemistry, University of Bath, Bath, UK

Improving reproducibility is a key challenge and topical area in the life sciences. The submitted manuscript provides a well written and interesting commentary on the topic and suggests two key approaches to improve reproducibility, based on technical review and incentivizing replication.

I think the paper is suitable for publication, but suggest the authors consider the following comments if they produce a revised version.

1. It is fair to praise Nature and EMBO’s recent efforts, but many scientists would put some blame for current problems on cut down methods sections, driven by space constraints which were/are imposed by some journals such as Nature and EMBO.
2. Standardised methodologies would need to be implemented carefully to avoid stifling scientific progress in developing methods and I suggest this would need to involve scientists as well as publishers?
3. I believe that clearer and longer methods sections are an important and easily achievable way to help improve reproducibility, we have written comments on one small aspect of this, the reporting of antibody use (Helsby MA, Fenn JR and Chalmers AD (2013) Reporting research antibody use: how to increase experimental reproducibility [v2; ref status: indexed, http://f1000r.es/1np ] F1000Research 2013, 2:153 (doi: 10.12688/f1000research.2-153.v2).  The authors mention the important RII, but I suggest they could give more prominence to the importance of comprehensive reporting of methods, controls and reagents. It would link directly to their point about better technical review as this is impossible without having well documented methods.
4. The section (2) on different ways to carry out replication could more specifically mention individual scientists trying to replicate findings for their own research, this work is already carried out so involves no additional funding. The key (as mentioned) is then incentivising scientists to publish this work.
5. I wonder what the authors think of initiatives like PubMed commons, aimed at collecting comments on papers, would this provide a format for shorter comments on the ability to reproduce key findings where the scientist concerned might not feel the data warranted a full publication? Is this another useful example of crowd-sourced post publication review?

I have read this submission. I believe that I have an appropriate level of expertise to confirm that it is of an acceptable scientific standard.
